# Supplementary material for: 3D printing with a 3D printed digital material filament for programming functional gradients
Source: Nat Commun. 2024 May 7;15:3605. doi: 10.1038/s41467-024-47480-5 (PMC11076495; doi:10.1038/s41467-024-47480-5)
Supplement: Supplementary file 1 — Supplementary Information [file 41467_2024_47480_MOESM1_ESM.pdf]

Supplementary Information

3D Printing with a 3D Printed Digital Material Filament for Programming Functional Gradients

Sang-Joon Ahn,[1,2] Howon Lee[2]* and Kyu-Jin Cho[1,2]*

[1]*Soft Robotics Research Center, Seoul National University, Seoul, Republic of Korea*

[2]*Department of Mechanical Engineering, Institute of Advanced Machines and Design, Seoul National University, Seoul, Republic of Korea*

*Corresponding author (e-mail: howon.lee@snu.ac.kr, kjcho@snu.ac.kr)

Table-of-contents for Supplementary Information

Supplementary Methods……………………………………………………………………3

Supplementary Figures…………………………………………………………………..8

Supplementary Figure 1. Extrusion ratio compensation for DM filament.

Supplementary Figure 2. DM filament design framework.

Supplementary Figure 3. A DM filament design framework.

Supplementary Figure 4. Effect of polymer mixing in a different composition.

Supplementary Figure 5. Uniaxial tensile test of CPLA-TPU blends with varying mixing ratios.

Supplementary Figure 6. 3-point bending test of CPLA-TPU blends with varying mixing ratios.

Supplementary Figure 7. DM filament designs with graded mixing ratio of two base materials.

Supplementary Figure 8. b-FDM 3D printed functional gradients using DM filaments.

Supplementary Figure 9. Design of the integrated circuit on the origami gripper.

Supplementary Figure 10. 3D Printing with DM filament using different 3D printers.

Supplementary Tables…………………………………………………………………..18

Supplementary Table 1: 3D printing parameters to build the DM filaments.

Supplementary Table 2: 3D printing parameters for 3D printing of target objects using DM filaments.

Supplementary Movies………………………………………………………………..…20

Supplementary Movie 1. b-FDM printing with a 3D printed DM filament.

Supplementary Movie 2. Comparison of b-FDM and conventional FDM.

Supplementary Movie 3. b-FDM printed multifunctional origami gripper.

Supplementary Movie 4. b-FDM printing with different commercial FDM printers.

Supplementary Methods

Extrusion ratio compensation for 3D Printing with DM filaments

Due to the discrete nature of the deposited layers, DM filaments have geometric deficiencies compared to commercial filaments, which may result in insufficient material extrusion. Thus, it is essential to compensate the insufficient extrusion of DM filaments during b-FDM printing of the target geometry (**Supplementary Fig. 1** and **Supplementary Table 2**). During b-FDM printing of the DM filaments, an extruder motor rotates and supplies the filament in an amount of E_f (**Supplementary Fig. 1a**). To prevent any under- or over-extrusions, E_f is generally set to be equal to the required amount of the filament to 3D print the target geometry, E_{tar}, that is, the extrusion ratio, E_f / E_{tar} =1. On the contrary, we increased the extrusion ratio to compensate geometric deficiencies in the DM filaments. To explore the effects of the extrusion ratio, a set of single-material DM filaments (composed of same PLAs) were 3D printed and extruded with different extrusion ratios to print the tensile test specimens (**Supplementary Fig. 1b-d**). Overall dimension of the specimen was set according to the ASTM D638-Type 4, while printing direction (nozzle movement direction) was set to be perpendicular to the extension. Three mechanical properties, namely, the weight, the ultimate strength, and the fracture strain, were measured as representative values. As a control group, specimens with E_f / E_{tar}= 1 were prepared using base filament, experimental results of which are plotted as a red star. The result show that mechanical properties of the b-FDM printed structures can be improved by increasing the extrusion ratio. The b-FDM printed specimens, with the extrusion ratio of the DM filament of 1.00, showed the weight of 1.93 g, the ultimate strength of 24.18 MPa, and the fracture strain of 9.07 %. These values are considerably lower than the results obtained using a commercial filament (2.01 g, 40.7 MPa, and 12.0%, respectively). Across all the experiments, b-FDM printed specimens with E_f

$/E_{tar}$= 1.05 exhibited properly improved properties which are comparable to the result of commodity filament. Increasing extrusion ratio to 1.10 results in even higher values, however, it is noteworthy that such over-extrusion may lead to additional defects such as blobs on the surface. Furthermore, we printed complex 3D geometry using commercial filament and DM filament with E_f/E_{tar}= 1.05 (**Supplementary Fig. 1e**). The b-FDM printed object exhibited the weight of 4.63 g and the height of 39.9 mm, which closely matches with those of the same object printed with the commercial filament having a weight of 4.66 g and a height of 40 mm. The results showed good agreement, confirming that the DM filament is comparable to commercial filaments.

DM filament design framework

We established a design framework to encode desired material property distribution of the target geometry (**Supplementary Fig. 2** and **3**). Firstly, target 3D geometry is decomposed (i.e. slicing) into a unit line representing a printing path (i.e. G-code command lines) through commercial slicers. By parsing the G-code, the printing parameters such as the printing sequence, the printing path, and the length of the supplied filament, E (with diameter D_f) are obtained. The programming resolution (i.e., size of the voxels in the target geometry) is determined by the longitudinal threshold on the DM filament (ΔE), which is theoretically defined by the size of the extrusion nozzle (0.4 mm). To achieve reliable programming and ensure sufficient transition between the dissimilar properties, we set this value to 2 mm or higher in our experiments. Therefore, a voxel in the 3D geometry is defined as a collection of multiple unit lines where the sum of their E values exceeds the ΔE. Each voxel with a specific material property is then mapped onto a specific location on the DM filament according to the printing sequence.

Subsequently, the material composition in a DM filament is spatially designed to achieve the desired material property at each voxel. These properties include color, tensional and flexural properties, and electrical conductivity, depending on the base materials used. With given materials, the concentrations in the volume fraction, $\{\phi\}$, and the interdigitated layer arrangement on the filament (represented by homogeneity parameter, η) are the key design factors. Users can select from presets whose resulting properties are given by experiment, or manually design with own parameters. In the case shown in **Fig. 1** and **Supplementary Fig. 2**, the color gradient is programmed by gradually varying the volume fraction of two base materials (represented with different colors, cyan and yellow). η is set to be 2 except for sections with a single material, since the perception of new colors can still be achieved despite localized aggregation of colors without the need for homogeneous mixing.

The printing schedule to build the DM filament is modified to minimize the frequency of switching between feedstock filaments (**Supplementary Fig. 3**). FDM 3D printing of vertically intersecting heterogeneous layers requires material switching between the layers. In general, FDM printers are programmed to conduct the material switching at every layer, which increases the printing time. The DM filament is divided into few homogeneous groups in a layer-by-layer manner, so that each group can be printed at once without material switching. This division occurs at the layer interface where heterogeneous layers intersect vertically. Consider the base material layer $L_{m,n}$, where m and n denote the base materials in the DM filament and the layer number, respectively. The grouping process begins with an ungrouped layer $L_{k,n}$, where k denote a specific base material in the DM filament. If $L_{k,n+1}$ exists and does not intersect with $L_{m\neq k,n}$, it will be added to the group. Note that if the intersection occurs, the printing material should be switched to deposit $L_{m\neq k,n}$ before $L_{k,n+1}$.

This process is repeated by increasing n until either the intersection occurs or $L_{k,n+1}$ does not exist. The result is a homogeneous group that can be printed without material switching. By conducting the same procedure for the remaining layers, the DM filament can be divided into a set of homogeneous groups. For the case shown in **Supplementary Fig. 2d**, the DM filament is divided into three groups (denoted by G_1~G_3). The printing process begins with yellow material inserted to the printer head, G_1 is printed at once without switching the material at every layer (**Supplementary Fig. 2d, i**). After the material switching, G_2 with heterogeneous material (marked with cyan) is deposited on G_1 (**Supplementary Fig. 2d, ii**). Finally, G_3 is printed after switching back to the yellow material (**Supplementary Fig. 2d, iii**). Thus, the DM filament, programmed to generate 13 color gradients, was successfully printed with just two material switches.

Based on the proposed design framework, we developed a custom-built filament design software using MATLAB. The software operates with following workflows: (i) import the G-code of the target object decomposed through commercial slicers; (ii) parse the G-code input to extract the printing sequence and the required amount of the filament for each voxel on the target object; (iii) program desired material property distribution on the DM filament by selecting from the presets or directly define material compositions and homogeneity parameters; (iv) generate and visualize the G-code for building the DM filament.

3-point bending test of b-FDM printed digital materials

Tunable electrical properties of b-FDM printed CPLA-TPU blends with varying volume fraction of TPU (ϕ_{TPU}) are further investigated through 3-point bending tests (**Supplementary Fig. 6**). We calculated the folding angle of specimens with thickness of 1.6 mm according to the ASTM D790 as follows:

$$\theta = 2asin\left(\left(\frac{L}{2}c + W(f-c)\right) \Big/ \left(\left(\frac{L}{2}\right)^2 + (f-c)^2\right)\right) \quad (4)$$

where θ, a, L, and f denote the folding angle, the thickness of the specimen, the span width between supports, and the displacement of the loading nose, respectively (**Supplementary Fig. 6a**). W and c can be expressed as $W = \sqrt{\left(\frac{L}{2}\right)^2 + (f-c)^2 - c^2}$ and $c = 2R + a$, respectively, with R which denotes the radius of the loading nose and supports. Flexural strength was measured at a break point or yield point of a load deflection curve.

The results showed that the flexural modulus (E_b) of printed structures can be easily modulated by controlling the material composition in the CPLA-TPU DM filament, varying over 20-fold from 73.3 MPa (TPU only) to 1598.5% (CPLA only) (**Supplementary Fig. 6b** and c). Moreover, the gauge factor ($GF = (\Delta R/R_0)\varepsilon_f$, where ε_f denotes applied flexural strain) of 3.69 is measured for TPU25 under flexural deformation, while CPLAs break up at bending of 79° with discrete, unstable electrical response (**Supplementary Fig. 6d**). A slight decrease in GF values is observed when ϕTPU is increased from 25% to 50%, where TPU50 exhibited the lowest flexural sensitivity with GF of 1.09. On the contrary, in the case of the TPU66 and TPU75, a slight increase in GF was observed (**Supplementary Fig. 6e**). We attribute this increase to the low content of CB particles, which could render more dispersive network, resulting in an increased resistance variation.

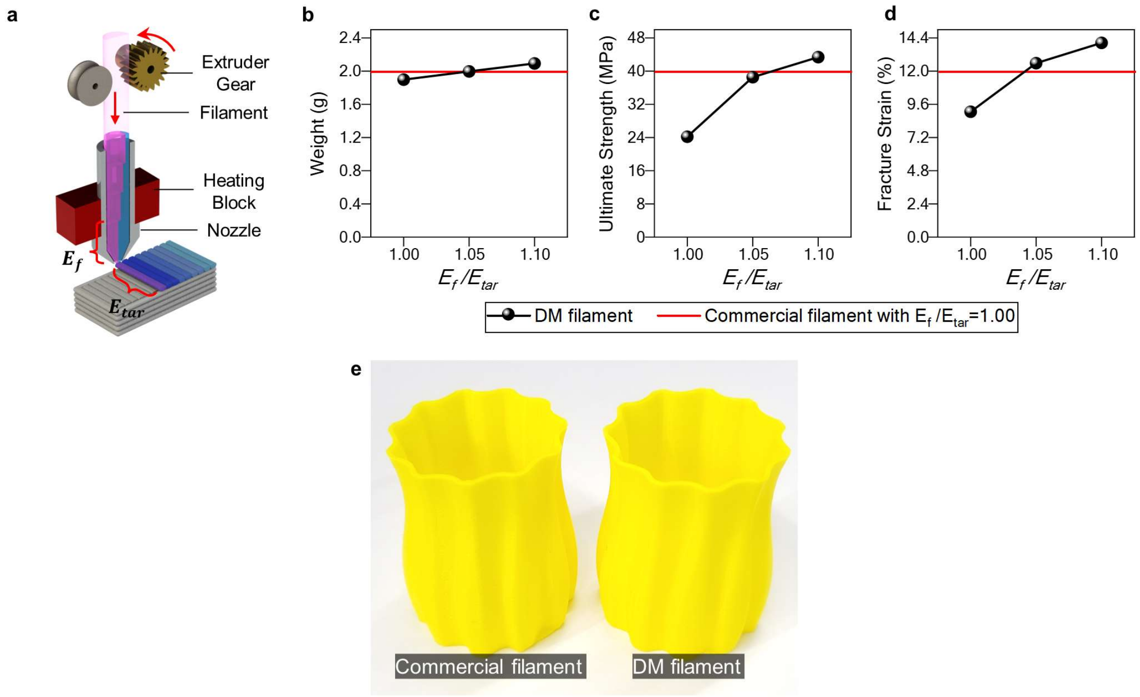

Supplementary Figure 1. Extrusion ratio compensation for DM filament. a Schematic of the extrusion process of DM filament. In typical FDM printing with a commercially available filament, supplied amount of the filament (E_f) is set to be equal to the required amount to build the target geometry (E_{tar}). **b** Weight, **c** ultimate strength, and **d** fracture strain of printed materials using DM filaments with different extrusion ratio (E_f/E_{tar}). Red lines indicate a control group printed using commercial filaments with E_f/E_{tar}= 1. **e** 3D object printed with commercial filament (left) and DM filament with E_f/E_{tar}= 1.05 (right). The results show that DM filament completely replaces its commercial counterpart with proper extrusion compensation.

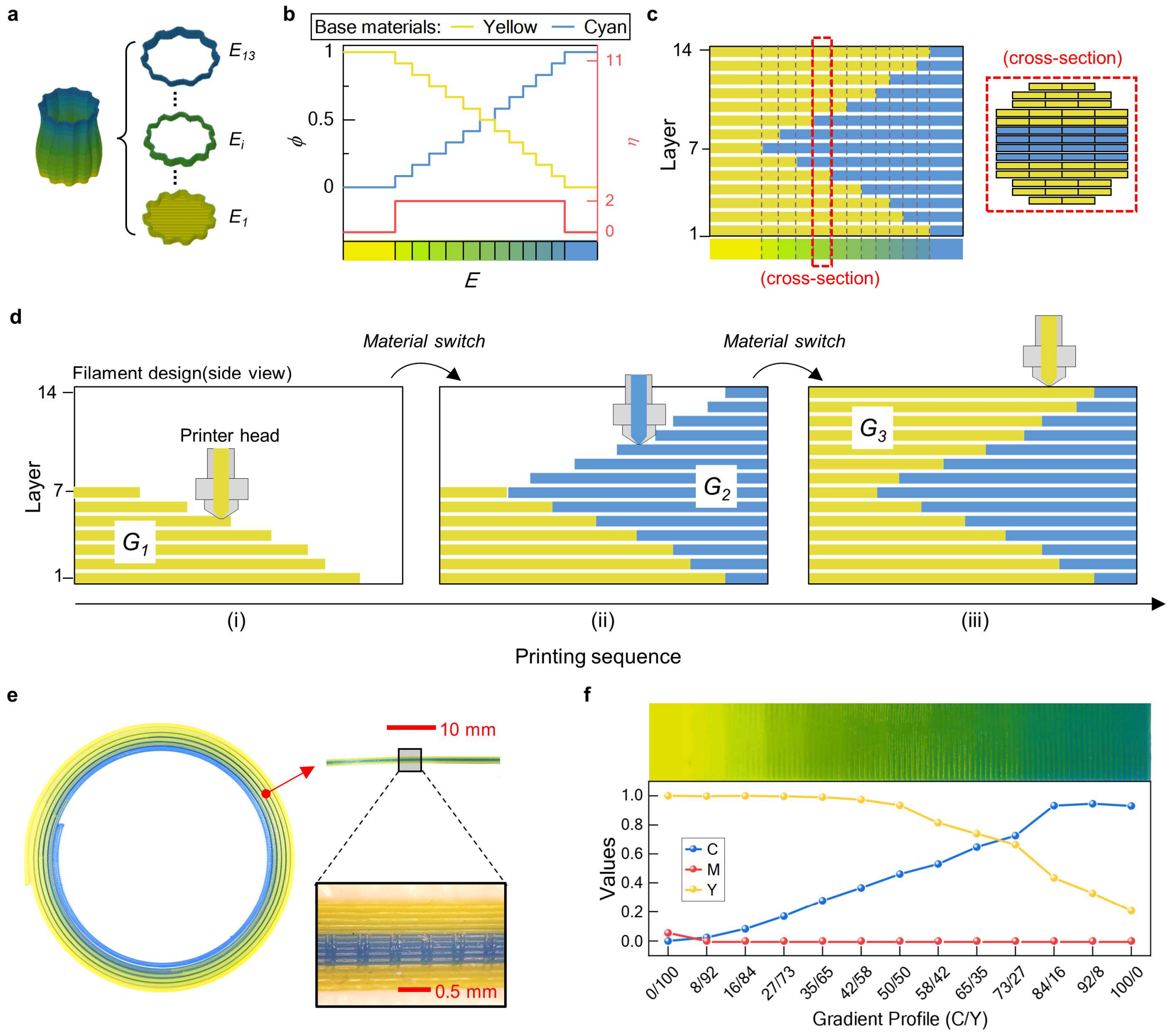

Supplementary Figure 2. DM filament design framework. a Voxelation of the target geometry. The target geometry is decomposed into a set of voxels with a required amount of the filament (E_i) and mapped onto the DM filament. **b** Spatial design of the material composition in the DM filament. The concentrations in the volume fraction, $\{\phi\}$, and the homogeneity parameter, η are the key design parameters to program desired material properties. **c** Constructed DM filament structure. **d** Printing schedule of the DM filament. By dividing the DM filament into three groups (denoted by G_1~G_3), the DM filament with 13 color gradient can be printed with only a two times of material switching. **e** 3D printed DM filament in the top view (left) and side view (inset). **f** Color gradient printed in a planar object (top) and measured CMY values (bottom).

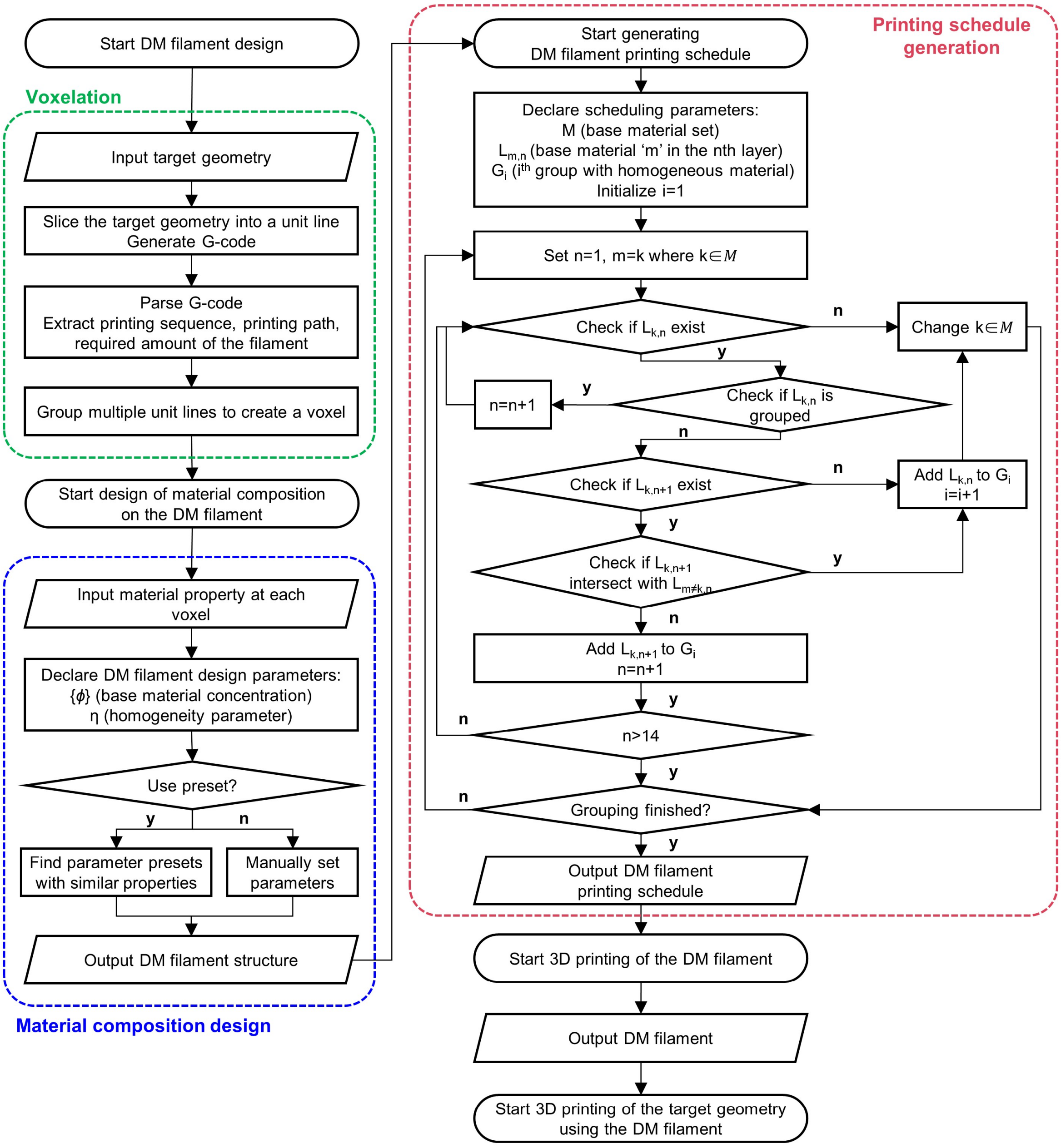

Supplementary Figure 3. A DM filament design framework. Target geometry is decomposed into a set of voxels which are defined as a collection of multiple unit lines. The material composition in the DM filament is designed to achieve target property distribution on the 3D geometry. Finally, a printing schedule for the DM filament is generated using a grouping algorithm.

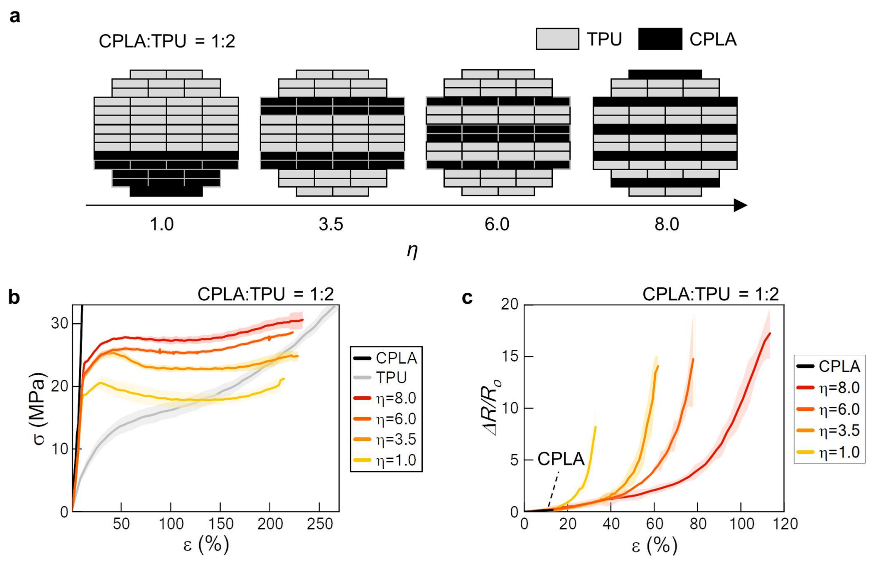

Supplementary Figure 4. Effect of polymer mixing in a different composition. a A set of CPLA-TPU DM filaments with varying η values ranging from 1 to 8, while maintaining a 1:2 ratio of the two materials. **b** Mechanical responses and **c** electrical responses during stretching of the specimens produced with DM filaments with varying η. The results indicate that our approach can be applied to different mixing ratios.

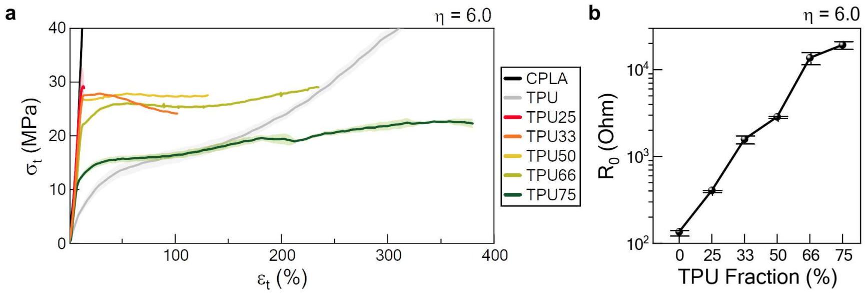

Supplementary Figure 5. Uniaxial tensile test of CPLA-TPU blends with varying mixing ratios. a Stress–strain curve of the different compositions under tension, illustrating the range of mechanical properties covered by mixing CPLA and TPU. **b** The initial resistance before stretching, R0, of tensile test specimens printed with different CPLA-TPU DM filaments.

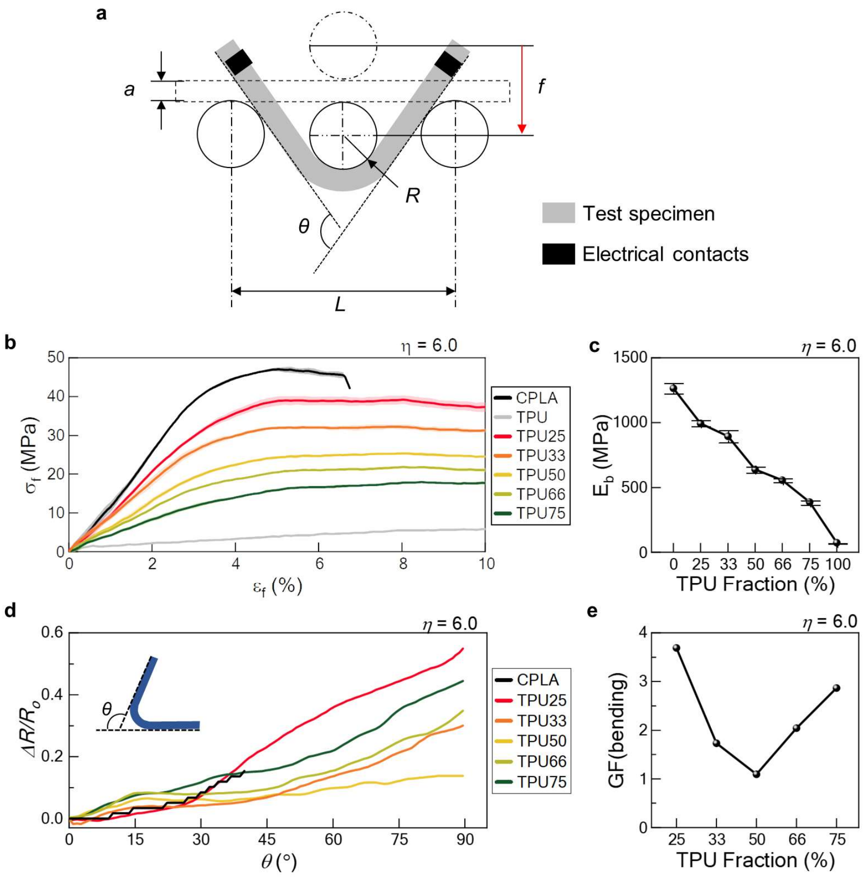

Supplementary Figure 6. 3-point bending test of CPLA-TPU blends with varying mixing ratios. a Schematic of the 3-point bending test. θ, a, L, f and R denote the folding angle, the thickness of the specimen, the span width between supports, and the displacement of the loading nose, the radius of the loading nose and supports, respectively. **b** Stress–strain curve of the different compositions during 3-point bending tests. **c** Flexural modulus as a function of TPU fraction. All error bars represent the standard deviation (n=5). **d** Representative electrical response of the different compositions. The electrical resistance change with respect to the base line resistance, $\Delta R/R_0$, during stretching was measured. **e** Gauge factor in response to bending as a function of TPU fraction.

a

	PLA	8/92	16/84	27/73	35/65	42/58	50/50	58/42	65/35	73/27	84/16	92/8	PETG
ϕ_{PETG}	0	0.08	0.16	0.27	0.35	0.42	0.5	0.58	0.65	0.73	0.84	0.92	1
ϕ_{PLA}	1	0.92	0.84	0.73	0.65	0.58	0.5	0.42	0.35	0.27	0.16	0.08	0
η	0	2	4	6.5	8	8	8	8	8	6.5	4	2	0
Cross-section													

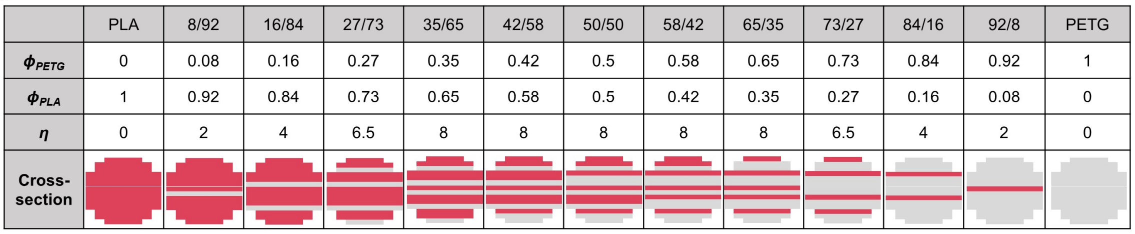

b

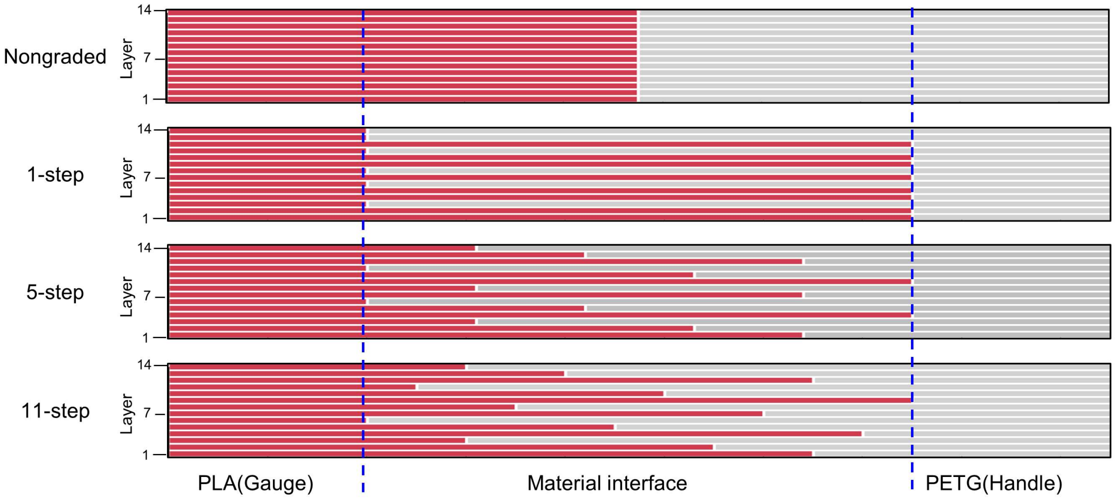

Supplementary Figure 7. DM filament designs with graded mixing ratio of two base materials. a Concentrations of base materials (top) and DM filament designs (bottom) with interdigitated layer arrangements. Each design yields the highest possible value of homogeneity parameters based on the given composition. **b** DM filament designs with different material profiles of at the interface.

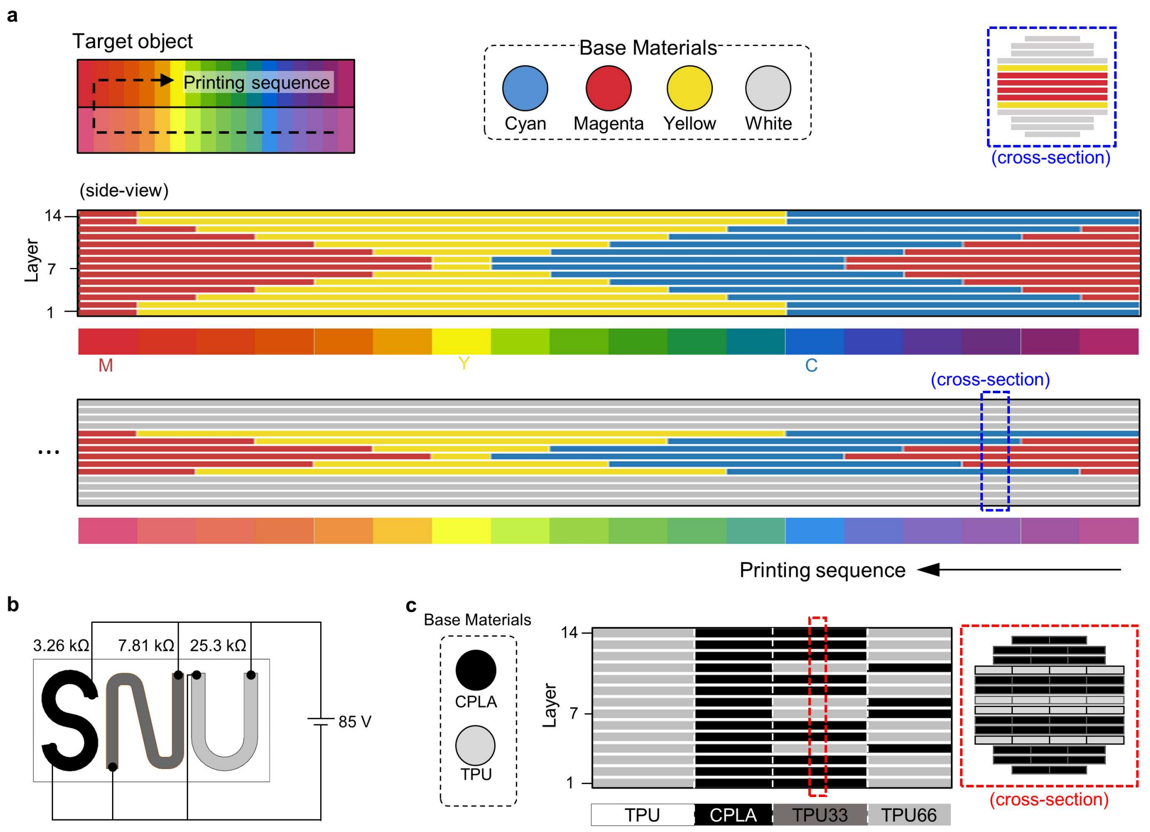

Supplementary Figure 8. b-FDM 3D printed functional gradients using DM filaments. a DM filament for full color 3D printing. Only four colors, cyan (C), magenta (M), yellow (Y), and white (W) are used to create 36 different colors. Each legend with its specific color was reconstructed using the measured RGB values of the printed object. **b** b-FDM printing of different levels of electrical resistivity onto the specific printing pattern and circuit design for Joule heating. **c** Design of the CPLA-TPU DM filament.

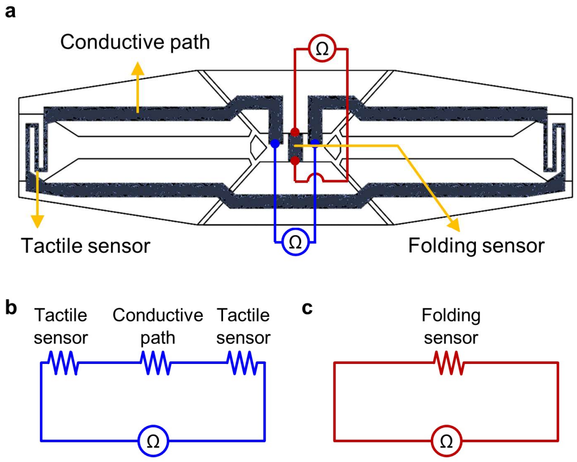

Supplementary Figure 9. Design of the integrated circuit on the origami gripper. a Schematic of the origami gripper. The blue and red lines indicate circuit diagrams for the tactile sensing and the folding sensing, respectively. **b** The circuit diagram for the tactile sensing where the tactile sensors are serially connected through the printed conductive path. **c** The circuit diagram for the folding sensing.

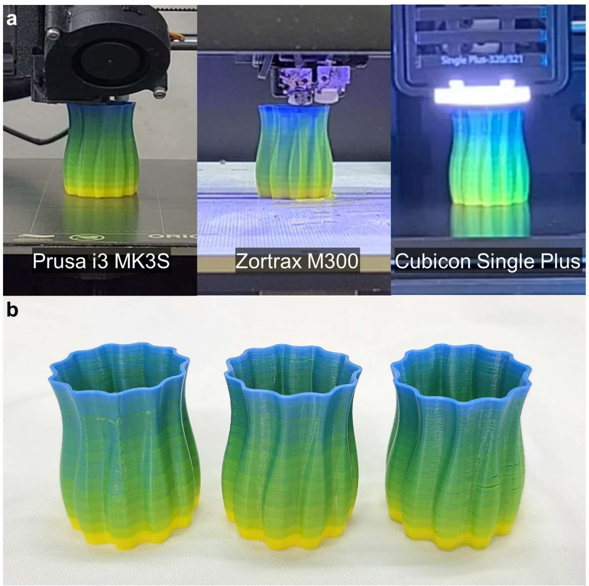

Supplementary Figure 10. 3D Printing with DM filament using different 3D printers. a 3D printing the target object with prescribed color gradient using three different FDM printers and same DM filaments. **b** Resulting 3D vases with color gradient. DM filaments can be readily adopted to any FDM printers, including the same printer used to print DM filament, to reproduce same results.

Supplementary Table 1: 3D printing parameters to build the DM filaments.

Feedstocks	PLA	PETG	CPLA	TPU
**Nozzle temperature (°C)**	220	240	220	235
**Bed temperature (°C)**	70	70	70	70
**Printing speed (mm/s)**	40	40	40	30
**Printing speed at first layer (mm/s)**	20	20	20	15
**Using cooling fan**	On	On	On	Off

Supplementary Table 2: 3D printing parameters for 3D printing of target objects using DM filaments.

Items	Nozzle temperature (°C)	Printing speed (mm/s)	Extrusion ratio (E_f/E_{tar})	Filament length (m)	DM filament printing time (h)	Target object printing time (h)
**3D vase with color gradient (Fig. 1)**	220	40	1.05	1.63	1.5	0.8
**Strain sensor (Fig. 2)**	235	30	1.1	0.66	<0.5	<0.5
**Mechanically graded specimen (Fig. 3)**	240	40	1.05	1.76	Depends on the type (<2)	1
**Color gradient (Fig. 3)**	220	40	1.05	2.18	2	0.5
**Origami gripper (Fig. 4)**	235	30	1.1	20.03	6	2.5
**'SNU' with thermal gradient (Supplementary Fig. 8)**	235	30	1.1	2.69	2.5	0.8

Supplementary Movie 1. b-FDM printing with a 3D printed DM filament.

A DM filament is first 3D printed using standard FDM printer and two base materials (standard filaments with different colors, cyan and yellow). When the DM filament is subsequently fed back to the same printer, the desired material gradient (13 levels of color gradient) appears in the target 3D object.

Supplementary Movie 2. Comparison of b-FDM and conventional FDM.

Two origami grippers with the same dimension but with the different spatial distribution of material properties are printed. The b-FDM printed origami gripper displays sharp folding along the creases without any delamination or mechanical failure. The conventional FDM-printed origami gripper without material gradient exhibited severe delamination during folding.

Supplementary Movie 3. b-FDM printed multifunctional origami gripper.

The b-FDM printed origami gripper with integrated electrical circuit demonstrates clear detection of the origami folding and contact to the grasped object. The folding sensor exhibited a consistent response upon folding, while the undesired signal from tactile sensors and the conductive path remained marginal. The tactile sensors produced a noticeable signal upon contact.

Supplementary Movie 4. b-FDM printing with different commercial FDM printers.

b-FDM 3D printing of the target object with prescribed color gradient using three different FDM printers and same DM filaments. The results are identical, indicating that the b-FDM with DM filaments can be readily applied to any standard FDM printers.
