# Supplementary material for: 3D printing with a 3D printed digital material filament for programming functional gradients
Source: Nat Commun. 2024 May 7;15:3605. doi: 10.1038/s41467-024-47480-5 (PMC11076495; doi:10.1038/s41467-024-47480-5)
Supplement: Supplementary file 3 — Description of Additional Supplementary Files [file 41467_2024_47480_MOESM3_ESM.pdf]

Description of Additional Supplementary Files

File Name: Supplementary Movie 1
Description: **b-FDM printing with a 3D printed DM filament.**

A DM filament is first 3D printed using standard FDM printer and two base materials (standard filaments with different colors, cyan and yellow). When the DM filament is subsequently fed back to the same printer, the desired material gradient (13 levels of color gradient) appears in the target 3D object.

File Name: Supplementary Movie 2
Description: **Comparison of b-FDM and conventional FDM.**

Two origami grippers with the same dimension but with the different spatial distribution of material properties are printed. The b-FDM printed origami gripper displays sharp folding along the creases without any delamination or mechanical failure. The conventional FDM-printed origami gripper without material gradient exhibited severe delamination during folding.

File Name: Supplementary Movie 3
Description: **b-FDM printed multifunctional origami gripper.**

The b-FDM printed origami gripper with integrated electrical circuit demonstrates clear detection of the origami folding and contact to the grasped object. The folding sensor exhibited a consistent response upon folding, while the undesired signal from tactile sensors and the conductive path remained marginal. The tactile sensors produced a noticeable signal upon contact.

File Name: Supplementary Movie 4
Description: **b-FDM printing with different commercial FDM printers.**

b-FDM 3D printing of the target object with prescribed color gradient using three different FDM printers and same DM filaments. The results are identical, indicating that the b-FDM with DM filaments can be readily applied to any standard FDM printers.
